# Classification of Two Volatiles Using an eNose Composed by an Array of 16 Single-Type Miniature Micro-Machined Metal-Oxide Gas Sensors

**DOI:** 10.3390/s22031120

**Published:** 2022-02-01

**Authors:** Jordi Palacín, Elena Rubies, Eduard Clotet, David Martínez

**Affiliations:** Robotics Laboratory, Universitat de Lleida, Jaume II, 69, 25001 Lleida, Spain; helenarubies@gmail.com (E.R.); eduard.clotet@udl.cat (E.C.); david.martinez@udl.cat (D.M.)

**Keywords:** electronic nose, eNose, MOX, principal component analysis, *k*-nearest neighbor

## Abstract

The artificial replication of an olfactory system is currently an open problem. The development of a portable and low-cost artificial olfactory system, also called electronic nose or eNose, is usually based on the use of an array of different gas sensors types, sensitive to different target gases. Low-cost Metal-Oxide semiconductor (MOX) gas sensors are widely used in such arrays. MOX sensors are based on a thin layer of silicon oxide with embedded heaters that can operate at different temperature set points, which usually have the disadvantages of different volatile sensitivity in each individual sensor unit and also different crossed sensitivity to different volatiles (unspecificity). This paper presents and eNose composed by an array of 16 low-cost BME680 digital miniature sensors embedding a miniature MOX gas sensor proposed to unspecifically evaluate air quality. In this paper, the inherent variability and unspecificity that must be expected from the 16 embedded MOX gas sensors, combined with signal processing, are exploited to classify two target volatiles: ethanol and acetone. The proposed eNose reads the resistance of the sensing layer of the 16 embedded MOX gas sensors, applies PCA for dimensional reduction and *k*-NN for classification. The validation results have shown an instantaneous classification success higher than 94% two days after the calibration and higher than 70% two weeks after, so the majority classification of a sequence of measures has been always successful in laboratory conditions. These first validation results and the low-power consumption of the eNose (0.9 W) enables its future improvement and its use in portable and battery-operated applications.

## 1. Introduction

Odor management has many practical applications for monitoring and quality supervision [1,2]. The measurement of the odor or aroma can be done with a panel of trained expert human operators, with a technical measurement of the individual chemical compounds or with an artificial olfactory system also called electronic nose, e-nose, or eNose.

Covington et al. [3] defined an eNose as an array of sensors that vary their selectivity according to the classes of chemical species analyzed. In general, this array is expected to include different types of sensors, which should have high sensitivity to a specific gas, rapid response time, a return to baseline levels, and high resistance to variable environmental conditions. However, the reality is that a sensor designed to have high sensitivity to a specific gas is also capable of detecting a variety of different gas and volatile compounds [4]. The effect of this cross-sensitivity is that there is no significant specificity in response to any gas although the appropriate combination of an array of sensors can be employed to maximize the number of relevant gases that can be incorporated into their response.

According to Wilson [5], an eNose can be used (1) to identify the source (plant, animal, or derived product) that produced a unique mixture of organic compounds (not the individual compounds) or (2) to assess one or more chemical, biological or physical characteristics for a specific purpose such as determining product consistency, quality, purity, age, or state of merchantability.

Covington et al. [3] presented a comprehensive recent revision of the state of the art of the concept of artificial olfaction in the 21st century, analyzing the recent applications of artificial olfaction based on the use of an eNose and comparing alternative gas sensing technologies: chemooptical [6], electrochemical [7] and chemoresistive [8]. Chemooptical devices are expensive and have big size, electrochemical sensors need maintenance, and chemoresistive technology usually has fast response and does not require maintenance, although having higher power consumption.

The wide variety of sensor technologies available to implement eNoses has led to the development of applications [9] tailored for diverse disciplines such as indoor air-quality monitoring [10,11], real time odor detection [12] and other applications such as environmental monitoring [13,14], food quality control [15,16], disease diagnosis [17,18], wood and paper processing, forest management, forest health protection, waste management and also agricultural sectors of agronomy [5] such as biochemical processing, botany, cell culture, plant cultivar selections, environmental monitoring, horticulture, pesticide detection, plant physiology and pathology.

This paper is specifically focused on the use and application of low-cost chemoresistive Metal-Oxide semiconductor (MOX) gas sensors, which is a technology widely used in arrays for gas sensing [19]. MOX gas sensors are based on a thin layer of silicon oxide with an embedded heater resistor that heats up the sensing material where the chemical interactions between the target gas and the metal oxide take place. The operating principle of MOX sensors is based on a reduction-oxidation (redox) reaction that affects the conductance of the metal-oxide layer. This reaction can be performed at different temperature set points that can affect the sensitivity of the sensor. The measurement of the presence and concentration of a gas is deduced form the measurement of the conductance of the metal oxide layer. MOX gas sensors usually have the disadvantage of crossed sensitivity to different gases and different sensitivities (variability) in each individual sensor unit. 

Chiu et al. [9] and Liu et al. [19] summarized the advantages and disadvantages of MOX sensors. The main advantages are high sensitivity, quick response to gas detection, short recovery times, limited sensing ranges, long lifetime and low cost. The main drawbacks include high temperature operation, high power consumption, poor precision and selectivity, drift in performance and limited sensor coatings. Moreover, the sensitivity of the sensing film is influenced by the temperature stability, humidity and background gas.

In the scientific literature there are some exhaustive papers focused on the description of the MOX gas sensors [20], on the analysis of the sensitivity and factors influencing MOX gas sensors [21], on the analysis of the gas-sensing properties of different metal-oxides [22], on the analysis of the individual performances of miniaturized MOX sensors according to their sensitizer material, target gas, metal oxide morphology, deposition process and type of heater [19], and on its application in environmental monitoring [13].

Finally, there are arising new promising gas sensor proposals. Ali et al. [23] reviewed the recent advances in the area of electrical mode sensors using organic small molecule n-type semiconductors based on perylene by highlighting its high sensing performance towards various volatile analytes. Choi et al. [24] have reported a process that addresses the main problems of chemical vapor deposition (CVD) graphene-based gas sensors by achieving high sensitivity and minimal variability as a gas sensor.

### New Contribution

According to Covington et al. [3], the production of miniaturized silicon MOX gas sensors has been the most remarkable improvement of MOX gas sensors over the last 10 years because this miniaturization reduces the power consumption and the response time.

The new contribution of this paper is the development of a new compact, low-cost and low-power eNose based on the use of 16 units of a single-type miniature silicon MOX gas sensor embedded in the commercial BME680 sensor device. In general, the most common disadvantage of any MOX gas sensor is its different volatile sensitivity and its different crossed sensitivity. These differences can become a very big problem for an application using only one MOX gas sensor. However, in this paper, the inherent variability and unspecificity of single-type MOX gas sensors, combined with signal processing, have been applied to create an eNose optimized to classify two target volatiles: ethanol and acetone. The proposed eNose reads the resistance of the sensing layer of the 16 embedded MOX gas sensors, applies PCA for dimensional reduction and *k*-NN for classification. The validation results have shown successful volatile classification of the two target volatiles in laboratory conditions.

## 2. Background

Table 1 presents a short reference list of scientific papers firstly presenting a specific eNose design based on an array of MOX gas sensors. As a summary, the majority of the papers listed in Table 1 propose the conventional development of an eNose based on an array of different sensor-types in which each type is sensitive to a specific gas: the number of MOX gas-sensors reported is between 2 and 96 with an average value of 15.6 and the sensor-types between 1 and 16 with an average value of 5.5.

As far as we now, Persaud et al. [25] presented in 1982 the first eNose using three MOX gas sensors with different sensitivities. The device analyzed odor quality and classified the types of odorants, including mixtures of odorous volatile compounds and single gases. This paper demonstrated that odor discrimination can be achieved without using highly specific receptors. The next paper highlighted in Table 1 was proposed in 1998 by Marco et al. [26] that proposed gas classification based on a self-organized map (SOM) [27] to identify different gases using an array of 6 MOX gas sensors. In close relationship with the objectives of this paper, in 2002 Arnold et al. [28] developed an array of 38 identical custom MOX gas sensors operating at different heating temperatures for air quality monitoring and early fire detection. This proposal was probably the first that demonstrated that an eNose created with a matrix of single-type MOX gas sensors can detect different volatiles. However, this proposal was based on custom MOX gas sensors that required a considerable amount of power to operate.

In the following decades different authors proposed a wide number of implementations combining different number and types of MOX gas sensors and applying different classification strategies. As far as we know, the next eNose reference paper using the same-type of MOX gas sensor was proposed in 2012 by Bennets [29], who used a matrix of 6 MOX gas sensors to detect and identify two gases. Lately, in 2014 Marco et al. [30] proposed the biggest eNose application combining 96 MOX gas sensors of 12 model types (with 8 units per sensor type) with 4 huge arrays of 4096 non-MOX resistive chemical gas sensors, that can be increased up to 65,536 gas sensors (16 arrays). Lately, in 2018 Burgués et al. [31,32] proposed two eNoses based on 12 and 7 MOX gas sensors of the same type for evaluating odor concentration.

Table 1 also presents the power spent by the eNose proposals. In general, this information is not described in the related papers as it was not considered relevant. This information was provided by Rossi et al. [33], but for the case of using a minimum array with 2 MOX gas sensors; by Palacín et al. [34], who used an eNose with 16 MOX gas sensors of 4 different sensor types with a total power consumption of 12.0 W, which was assumable for a medium size mobile robot application; and by Burgués [35], who proposed a portable eNose with 16 MOX gas sensors of 4 different types carried in an unmanned aerial vehicle (UAV) operating as a drone and using an operation strategy that reduced the average power consumption to 1.0 W. Finally, the power consumption of the custom eNose proposed in this current paper is 0.9 W in continuous gas sampling operation because of the low-power requirements of the commercial MOX gas sensors used.

**Table 1:** List of papers firstly presenting an eNose using MOX gas sensors.

Year	Reference	V (V)	I (A)	P (W)	MOX		Other non-MOX		Classification Method	Volatiles Detected
					Number	Types	Number	Types		
	*This paper*	5	0.18	0.9	16	1	0	0	PCA [36]-*k*NN	2
2021	Burgués [35]	-	-	1.0	16	4	5	2	PLSR [37]	1 ^A^
2020	Arroyo [38]	5	0.19	0.9	4	4	0	0	NN [39]	2^C^
2020	Burgués [40]	-	-	-	27	5	0	0	None	1 ^C^
2020	Tiele [41]	12	-	-	10	10 ^G^	1	1	PCA [36]	3 ^C^
2019	Palacín [34]	12	1.0	12.0	16	4	0	0	PLS-DA [42]	2
2019	Fan [43]	-	-	-	2	2	3	3	OCGM-OCNN	3 ^C^
2018	Burgués [31]	-	-	-	7	1	0	0	None	1 ^C^
2018	Burgués [32]	-	-	-	6 + 3+3 ^D^	1	0	0	PLS [44]	1 ^C^
2018	Gongora [45]	-	-	-	6	5	1	1	DNN	10
2017	Monroy [46]	-	-	-	10	8	0	0	PCA-SVM	2
2016	Rossi [33]	-	-	2 × 0.76	2	2	0	0	Threshold	1 ^A,C^
2016	Schleif [47]	-	-	-	5	5	0	0	SGTM-TT	4
2016	Fonollosa [48]	-	-	-	5 × 8	4	0	0	SVM [49]-SVR	4 ^C^
2015	Vries [50]	-	-	-	5 × 4	-	0	0	PCA-ANOVA	4
2015	Westenbrink [4]	-	-	-	8 ^G^	-	3	2	LDA [51]-*k*NN	3
2015	Fonollosa [52]	-	-	-	16	4	0	0	RC [53]	2 ^A,E^
2014	Marco [30]	-	-	-	96	12	4 × 4096	31	PCA [36]-PLS	2
2014	Rossi [54]	-	-	0.130	8 ^G^	-	-	-	-	-
2014	Sanchez [55]	-	-	-	8	4	0	0	None	1 ^A^
2014	Monroy [56]	-	-	-	7	7	0	0	Kernel DM+V	1 ^A^
2014	Bennetts [57]	-	-	-	3	3	0	0	PCA [36]	2
2013	Savarese [58]	-	-	-	10	-	0	0	PCA [36]	2 ^F^
2013	Monroy [59]	-	-	-	11	9	0	0	Regression	1 ^A^
2012	Vergara [60]	-	-	-	16	4	0	0	SVM	6 ^C^
2012	Bennetts [29]	-	-	-	6	1	0	0	MV RV M [61]	2
2012	Aguilera [62]	-	-	-	16	16 ^G^	0	0	ICA [63]-PLS-ANN	15 ^F^
2012	Brudzewski [64]	-	-	-	2 ^B^ × 12	8	0	0	PCA-SVM [49]	5, 11 ^F^
2011	Haddi [65]	-	-	-	6	6	0	0	PCA-SVM [49]	5 ^F^
2011	Gonzalez [66]	-	-	-	4 ^B^ × 7	7	0	0	None	1 ^A^
2010	Brudzewski [67]	-	-	-	2 ^B^ × 12	8	0	0	2D convolution	6 ^F^
2010	Guo [68]	-	-	-	12	12	0	0	PCA [36]-*k*NN	4 ^F^
2010	Mildner [69]	-	-	-	3 × 6	-	0	0	2 × PCA [36]-PLS	3 ^F^
2009	Lilienthal [70]	-	-	-	6	5	0	0	Kernel DM + V	1 ^C^
…										
2002	Arnold [28]	-	-	-	38	1 ^G^	0	0	PCA-LDA [51]	2 ^E^, 1 ^A^
…										
1998	Marco [26]	-	-	-	6	3 + 3 ^G^	0	0	SOM [27]	6
…										
1982	Persaud [25]	-	-	-	3	3	0	0	-	E, F

^A^ Detecting the overall odor concentration or the overall concentration of volatile substances. ^B^ Using arrays operating differentially. ^C^ Estimating gas concentration. ^D^ Using different power management strategies. ^E^ Detecting a mixture of volatiles. ^F^ Estimating the mixture of volatile compounds. ^G^ Customized sensor.

## 3. Materials and Methods

The materials used in this this paper are the BME680 MOX gas sensor, a custom array of 16 MOX gas sensors based on the BME680, and two target gases: ethanol and acetone. The methods used in this paper to classify the readout of the array of gas sensors are the principal component analysis (PCA) and the k-nearest neighbor algorithm (*k*-NN).

### 3.1. BME680 Sensor

**Figure 1:**
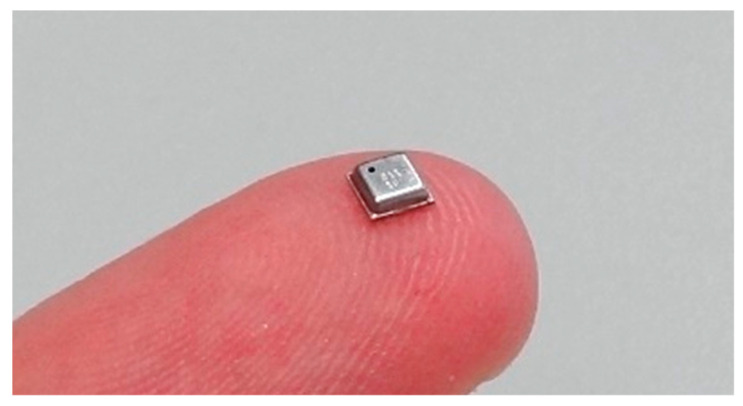
The BME680 sensor.

Figure 1 shows the BME680 sensor used in this paper, manufactured by Bosh Sensortec (Reutlingen, Germany). The BME680 is a digital miniature sensor able to measure air quality, temperature, humidity and pressure. The BME680 includes an internal miniature MOX gas sensor to externally estimate the air quality. This eNose proposal is based on reading the resistance of the sensing layer of the embedded MOX gas sensor, so the BME680 is used as a kind of digital MOX gas sensor. This paper then exploits the inherent variability and unspecificity that must be expected from this embedded MOX gas sensor to implement an eNose tailored to classify two target volatiles.

The BME680 digital miniature sensor is encapsulated on a small and compact metallic LGA package with a footprint of 3.0 × 3.0 mm and a height of 1.00 mm (Figure 1). The BME680 is a low-power digital 4-in-1 micro-electromechanical system (MEMS) sensor, which is able to simultaneously measure temperature, pressure, humidity and indoor air quality. The BME680 includes integrated circuits for signal conditioning and bus serial communication, and can be externally accessed by using the I^2^C and the SPI serial bus. The BME680 operating as a gas sensor is proposed and optimized to unspecifically detect volatile organic compounds (VOCs) in indoor air quality management applications. The MOX gas sensor embedded in the BME680 measures the conductance change of the metal-oxide layer caused by the adsorption of total volatile organic compounds (TVOC) polluting the air (except CO_2_). The MOX gas sensor of the BME680 is not selective or specific, so the effect of a specific gas is not distinguished from the others. Finally, the information gathered by the BME680 can be externally processed in order to estimate an indoor air quality (IAQ) index in a scale range from 0 (clean air) to 500 (heavily polluted air).

#### 3.1.1. Operation of the BME680 Sensor

**Figure 2:**
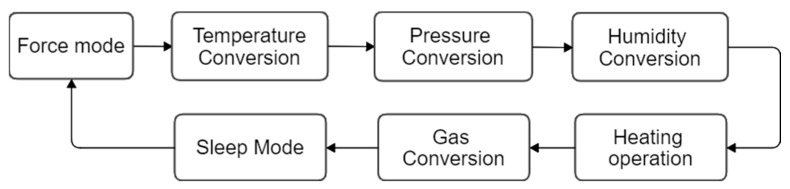
Sequence of ADC conversion and gas sensor heater operation.

The BME680 sensor supports two operation modes: Sleep and Forced mode. The sensor automatically operates in Sleep mode after a power-up sequence. In Sleep mode no measurements are performed and the power consumption is minimum. When the device operates in Forced mode the sensor starts a measurement sequence to get the temperature (T), pressure (P), humidity (H) and gas resistance (G) and then operates automatically in Sleep mode (see Figure 2). The BME680 has internal registers to control the effective measurement of the TPHG values and it can be configured, for example, to measure only temperature (T) or only temperature and humidity (TH) or only gas (G). This paper is only focused on the gas sensor readout (G) obtained from the sensor. The real duration of this TPHG measurement sequence depends on the configuration of the oversampling values of the temperature, pressure, humidity sensor and the duration of the heat up defined to complete a gas sensor measurement.

#### 3.1.2. Configuration of the BME680

The BME680 has a set of control registers that must be used to configure the main parameters of the sensor: *Config*, *Ctrl_meas*, *Ctrl_hum*, *Ctrl_gas_1*, *Ctrl_gas_0*, *Gas_wait_x*, *Res_heat_x*. The operation mode (Sleep or Forced) is defined in the two least significant bits of the control register *Ctrl_meas<1:0>*. The values of the configuration parameters that define the behavior of the BME680 are summarized in Table 2. These parameters allow: (1) skipping or defining a sampling time for the measurement of temperature (T), pressure (P) and humidity (H) and setting the oversampling value; (2) configuring the length of the IIR filter coefficient used to filter and remove short-term fluctuations on the temperature and pressure measurements; (3) configuring the gas (G) measurement procedure by defining four parameters: enable heater, enable gas conversion, setting the heat up duration (a value that codes a range from 1 to 4032 ms) and setting the target heater temperature (a value that codes a range from 200 to 400 °C). 

#### 3.1.3. Measurement of the Resistance of the Sensing Layer of the MOX Gas Sensor

The use of the MOX gas sensor embedded in the BME680 requires three steps: heating the gas sensor hot plate at a defined target heater temperature (the typical values are between 200 °C and 400 °C); maintaining this temperature during the defined heat up duration (the typical value is 100 ms); and finally measuring the resistance of the gas sensitive layer of the MOX gas sensor.

The final determination of the value of the resistance of the gas sensing layer requires the readout of the ADC values stored on two internal registers: *gas_r_lsb<7:6>* and *gas_r_msb<7:0>*. The resolution of the gas ADC value is 10 bit. The conversion of the ADC value into a resistance expressed in ohms is determined by the following equation provided by the manufacturer:

var1 = (1340.0 + 5.0 · *range_sw_err*) · const_array1[*gasRange*] (1)

gas_res = var1 · const_array2[*gasRange*]/(*gas_adc* − 512.0 + var1) (2)

where *range_sw_err* is an internal configuration parameter that must be read from the register address 0x04 <7:4> (signed 4 bit). The *gasRange* is another internal configuration parameter that must be read from the register *gas_r_lsb<3:0>* in order to determine the constants required to compute the resistance. The *gas_adc* is the ADC value read from the *gas_r_lsb<7:6>* and *gas_r_msb<7:0>* registers. The list of possible values of the register *gasRange* and the corresponding array values of const_array1 and const_array2 arrays are provided in the datasheet of the sensor and summarized on Table 3. 

As a summary, the determination of the value of the resistance of the sensing layer of the MOX gas sensor (gas_res) requires the reading of: *range_sw_err*, *gasRange*, *gas_adc*, and the use of the vector of constants defined in const_array1 and const_array2. 

### 3.2. eNose as an Array of 16 Single-Type BME680 Gas Sensors

**Figure 3:**
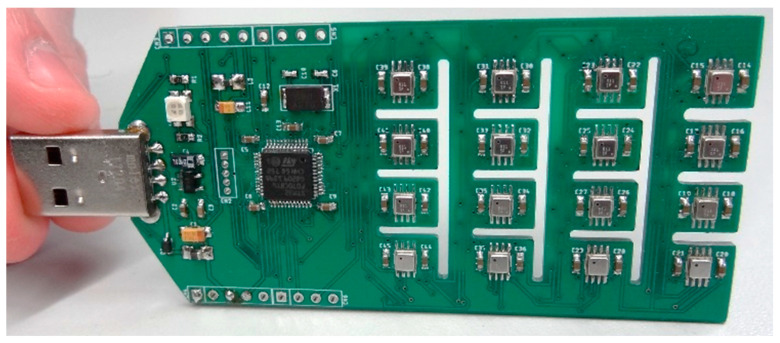
eNose used in this paper: detail of the PCB and of the array of 16 BME680 sensors.

Figure 3 shows the eNose presented in this paper, which is based on a single Printed Circuit Board (PCB) (80.87 mm × 40.01 mm) designed with Autodesk Eagle software. The PCB includes holes to facilitate air circulation around the gas sensors. This electronic board has been designed to be powered from two redundant sources: the main USB connector, which provides 5.0 V, and an additional power connector designed to plug a redundant or backup power to maintain the eNose activated after powering off the main USB connector. The 5.0 V are converted to the internal 3.3 V used by the electronic devices through a voltage regulator (LD3985M33R). The board also includes different LEDs for status information and a Serial Wire Debug (SWD) interface to program the microcontroller.

The main Micro Controller Unit (MCU) is the STM32F070CBT6 from STMicroelectronics, which is a low-power version of the high-performance ARM Cortex-M0 32-bit RISC (Reduced Instruction Set Computing) core operating internally at 48 MHz by using an external 8 MHz crystal oscillator. This MCU incorporates 128 Kbytes of Flash memory and 16 Kbytes of SRAM in a small surface mount LQFP48 package. This MCU offers standard serial communication interfaces (I2Cs, SPIs, and USARTs), one USB 2.0 Full-speed interface, one 12-bit ADC and several general-purpose 16-bit timers. This MCU was selected because it was the smallest low-power STM32 microcontroller capable to handle the USB and SPI communication interfaces simultaneously.

The communication with the array of BME680 is performed using the SPI serial bus. The MCU operates as a SPI master and the 16 BME680 sensors operate as slaves through a multi-slave SPI topology where the SCLK, MOSI and MISO lines of the SPI communication are shared between all the sensors. The SPI communication with a specific BME680 chip is controlled with the chip select line (CSB line). This configuration allows to obtain the data from an array of BME680 sensors by using a single SPI peripheral bus (using the SCK, SDI and SDO lines) from the MCU but requires the use of 16 dedicated digital lines to individually select the CSB line of a specific sensor BME680 sensor.

As stated in the background section, the average number of MOX gas sensors used in the arrays listed in Table 1 is 15.6, but the decision to use 16 sensors in the eNose does not have a scientific motivation. In this case, the small MCU selected was not able to handle 25 sensors using a wire selection, so this number was practically limited to 16.

The USB connection of the electronic board of the eNose has been designed to operate as a slave USB device controlled by an external master USB device such as a Personal Computer, an Arduino, a RaspberryPI, an additional control board, etc. The USB communication is a USB-CDC software-based serial port (software compatible with the old RS-232 protocol) operating as a Virtual Com port. This configuration simplifies the integration of the eNose in a general measurement framework.

Finally, the maximum power consumption of the complete eNose device is 0.9 W (5.0 V and 180 mA) when continuously measuring the gas sensor readout with the BME680 sensors operating in Force mode at the maximum heating temperature, and only 0.05 W (5.0 V and 10 mA) whit the BEM680 sensors in Sleep mode.

#### 3.2.1. Individual Configuration of the Array of 16 BME680 Gas Sensors

Table 4 details the configuration of the individual parameters that must be set or defined in order to perform a gas measurement with a BME680 sensor. The main parameters that define a gas measurement are the Heat up duration and the Target heater temperature. Table 5 shows the individual values assigned in this paper to the array of 16 BME680 sensors: a common value of 150 ms for the Heat up duration and a lineal range from 200 °C to 400 °C for the Target heater temperature. This array configuration is proposed just as an initial (trial) selection with the expectation that the different heater temperatures combined with the inherent MOX gas sensors variability will provide different sensitivities to individual gases. The number of possible combinations is huge but this proposal is in concordance with the linear range of power or PWM applied to the same type of MOX gas sensors used in other conventional eNoses [34]. A detailed analysis focused in the determination of an optimal configuration of the array of gas sensors will be performed in future analysis.

#### 3.2.2. eNose Normal Measurement Operation

**Figure 4:**
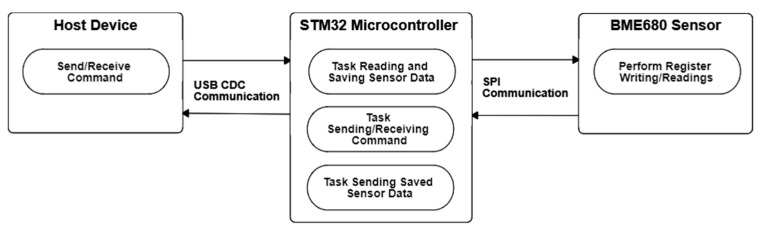
Diagram of the main sequential tasks and exchange of information between devices.

The eNose is in a normal measurement mode after power up. Figure 4 shows the three main tasks developed by the MCU in a normal measurement operation. The first task consists on a non-blocking continuous measurement procedure that sequentially reads the temperature, pressure, humidity and gas resistance of the sixteen BME680 sensors at the maximum velocity provided by the SPI serial bus and allowed by the sensors. The data gathered are stored internally on a SRAM buffer in order to provide fast response upon external demand. The second task receives the USB-CDC commands from the host device and provides specific answers if needed. The commands allow the configuration of all eNose parameters and can be used to request the current gas sensor measurements internally stored. The third task is activated by a specific host command and is in charge of continuously submitting the gas sensor measurements to the USB host at a requested interval. This feature is very interesting in order to simplify the automatic analysis of the data gathered by the eNose, which can be automatically implemented in the host USB as an isolated procedure activated upon receiving this periodic submission [34].

### 3.3. Target Gases Used to Train and Test the eNose

The target gases used in this paper to validate the array of single-type miniature MOX sensors are ethanol and acetone. These two gases have been widely used in the scientific literature [34,43,46]. Both gases are liquid in their standard state, which is used in many industrial and academic activities, but they evaporate quickly and thus can appear as volatiles in a human workspace as a consequence of an accidental leakage. 

### 3.4. Reference Measurements of Volatile Concentration

In this paper, the measurement of the real concentration of the volatiles analyzed is performed with a compact photo ionization detector (PID) ppbRAE 3000, manufactured by RAE Systems. This PID provides a fast and true concentration measurement of a known gas or chemical compound. However, the target chemical compound (up to 200 compounds) must be correctly configured in the device because a PID has no classification capabilities as it only provides a concentration value corresponding to the mixture of all present gases with an ionization energy below what its ionization unit delivers [59]. Therefore, the concentration measured with a PID is not useful when the gas or chemical compound measured is unknown.

### 3.5. Principal Component Analysis (PCA)

The principal component analysis (PCA) proposed by Pearson [71] is the process of computing the principal components of a collection of points and using them to change the basis of the data [36]. The result of the PCA are the eigenvectors of the covariance matrix of a collection of points. In this paper, PCA is used to reduce the dimension of the data gathered from the eNose while preserving as much of the variation of the data as possible.

### 3.6. k-Nearest Neighbors (k-NN)

The *k*-nearest neighbors algorithm (k-NN) proposed by Fix and Hodges [72] is a non-parametric classification method that finds the k-closest data samples in a data set describing different classes and determines a classification membership according to the plurality vote of the classes of the *k*-closest nearest neighbors. The class membership is the most common class among its *k*-nearest neighbors. If *k* = 1, then the object is simply assigned to the class of that single nearest neighbor. In this paper, *k*-NN is used to classify the PCA projection of the current eNose data sample using as a reference data set the PCA projection of the results of the calibration experiments. The final objective of *k*-NN will be the classification of the current eNose data sample into ethanol or acetone.

## 4. Calibration of the eNose with Ethanol and Acetone

This section shows the calibration of the proposed eNose in order to detect two target gases: ethanol and acetone. This calibration is inspired in [34], in which a custom eNose based on conventional MOX gas sensors was embedded in a mobile robot in order to early detect gas leaks of the similar target gases. Therefore, the goal of this calibration is to obtain static evaporation profiles similar to the dynamic evaporation profiles analyzed in [34]. The result of this calibration will allow the future application of the eNose proposed in this paper in similar early detection applications.

The calibration experiments are based on using a heater of 60 W to force the evaporation of liquid acetone and ethanol during 250 s. During this calibration experiments the eNose and the PID are placed above the evaporator at a relative height of 0.25 m. The eNose and the sample tube of the PID are placed together to measure the convective currents generated by the evaporator.

**Figure 5:**
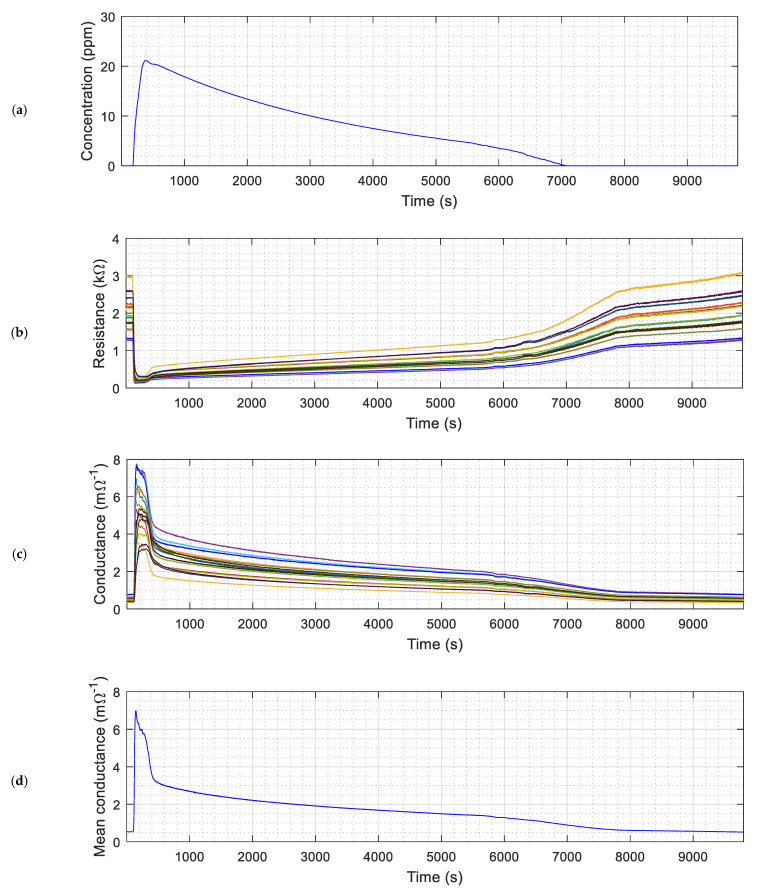
Evolution of the sensor readings obtained during an experiment with ethanol: (**a**) Ethanol concentration measured with the ppbRAE3000 sensor; (**b**) Evolution of the raw resistance of the 16 MOX sensors; (**c**) Evolution of the conductance of the 16 MOX sensors; (**d**) Evolution of the mean conductance of the 16 MOX sensors.

Figure 5 shows the raw measurement results obtained with the eNose and the PID. Figure 5a shows the evolution of the concentration of ethanol measured with the PID that is provided just as a reference. The concentration of ethanol suddenly increases when power is applied to the evaporator heater and slowly decreases when power is removed and forced evaporation ends. Figure 5b shows the raw evolution of the resistance of the sensing layer of the 16 MOX gas sensors of the eNose. This resistance suddenly decreases as a consequence of the presence of evaporated ethanol. Unexpectedly, the response of the MOX gas sensors to the presence of ethanol in air is faster than the PID, probably because the circulation of a forced convection. Figure 5c shows the deduced evolution of the conductance and Figure 5d shows the evolution of the average conductance of the 16 MOX gas sensors of the eNose. This average conductance is used in this paper to binary detect the presence of gas by the application of a threshold value relative to the background conductance level obtained in clean air conditions.

This calibration experiment has been repeated evaporating acetone obtaining very similar transitory results to those obtained when evaporating ethanol.

**Figure 6:**
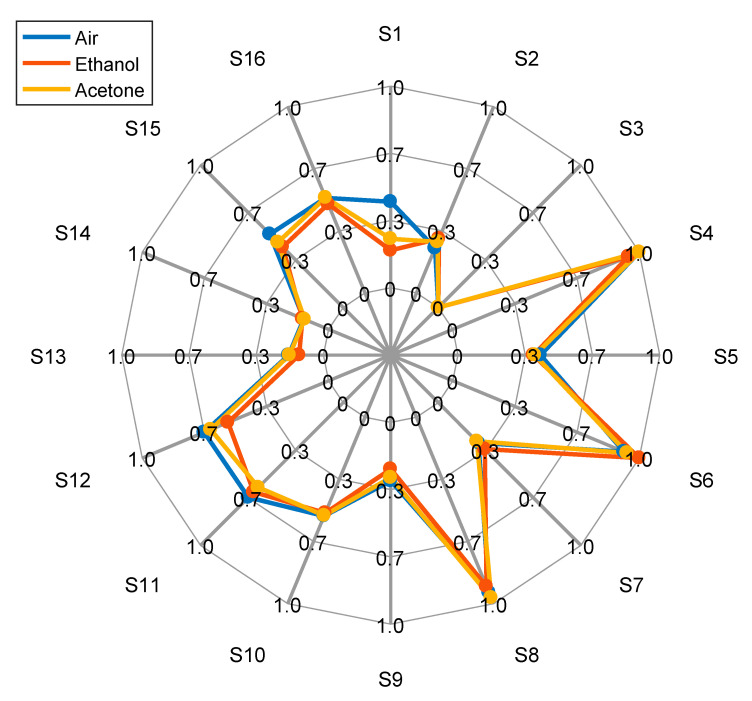
Normalized representation of the maximum conductance values when the sensors are exposed to air (blue), ethanol (orange) and acetone (yellow).

Figure 6 shows the normalized radial representation of the maximum conductance measured with the sensors of the eNose in the presence of ethanol (orange line), and acetone (yellow line). Figure 6 also shows the average conductance measured in the case of clean air (blue line). Figure 6 shows small differences between the ethanol and acetone profiles, which may allow the classification of both gasses. Specifically, the sensor that offers the maximum difference between the ethanol and acetone cases is S12, heated at 350 °C, which is the recommended operation temperature of the MOX gas sensor embedded into the BME680.

**Figure 7:**
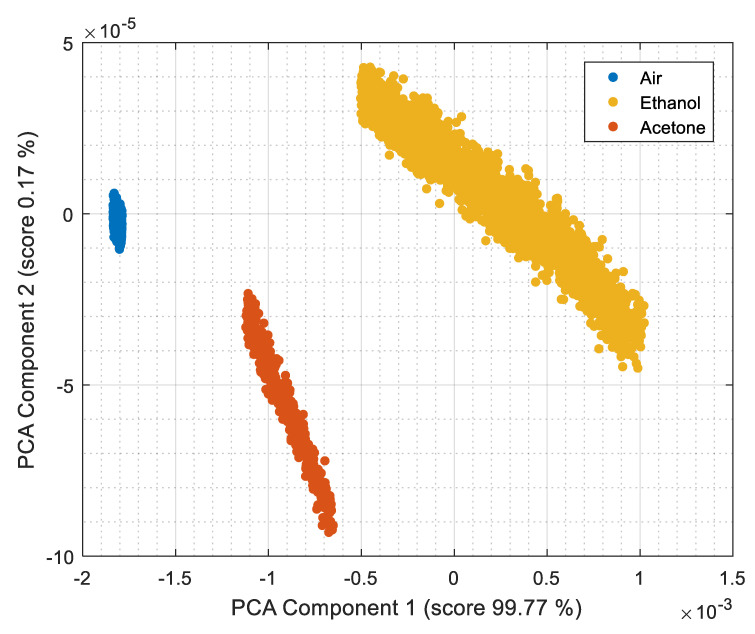
PCA for air, ethanol and acetone.

Figure 7 shows the results of a PCA analysis applied to the raw conductance information measured from the 16 MOX gas sensors embedded in the 16 BME680 used in the eNose presented in this paper. Figure 7 shows the first and second principal components proposed by the PCA analysis of data measured for the cases of forced evaporation of ethanol (yellow) and acetone (red) (see Figure 5 and Figure 6). The first component scores the 99.77% of the variation of the conductance of the MOX gas sensors and the second component scores the 0.17% of this variation. The first component is truly representative of the variation of the measurement, but the use of two components facilitates the visual interpretation of the results. After completing the PCA using the reference samples of ethanol (yellow) and acetone (red) we have applied the PCA projection to general air measurements (blue) just to observe its location in the projected space. Figure 7 shows these three classes (ethanol, acetone and air) clearly differentiated in the projected space.

In this paper, we apply a threshold to the variation of conductance to detect a variation in the concentration of volatile substances. The relative conductance threshold was defined as 0.6 mΩ after a trial and error procedure. This means that the eNose detects the existence of a volatile substance when the average conductance increases 0.6 mΩ from a background reference value that has been obtained by averaging this average conductance during a time window of 300 s. At this stage, this background reference value is constantly updated until the detection of a gas, as we usually end the experiments after detecting a gas.

The use of this detection threshold is in part responsible of the good scores of the two principal components shown in Figure 7. This is because the application of the threshold potentially avoids the classification of conflictive measurement points obtained in the cases of very low gas concentrations and very low and similar conductance values. Therefore, the use of a detection threshold simplifies the PCA analysis and contributes to separate the gas classes. 

## 5. Validation of the eNose to Detect Ethanol and Acetone

**Figure 8:**
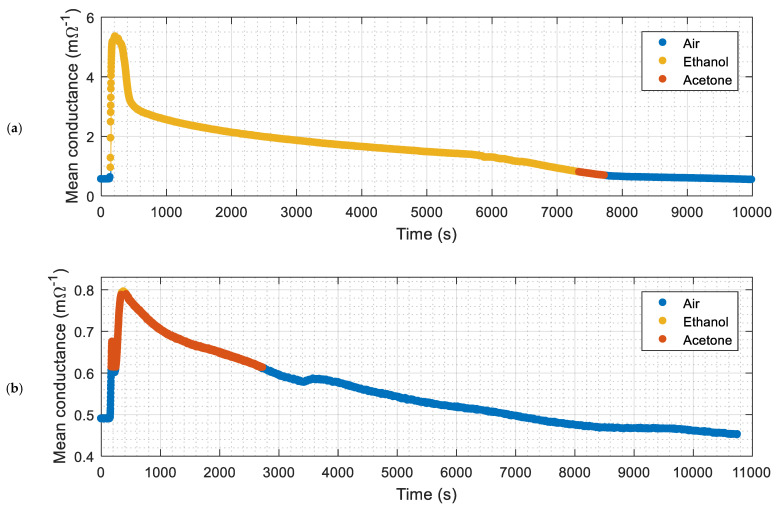
Evolution of the classification results obtained during two different experiments in the presence of: (**a**) Ethanol; (**b**) Acetone.

The ethanol and acetone projections shown in Figure 7 are used as a reference dataset for a *k*-NN classification (with *k* = 5). Figure 8 shows the instantaneous classification results obtained in two validation experiments conducted with (a) ethanol and (b) acetone. Figure 8 shows the evolution of the average conductance measured by the eNose and the result of the volatile classification labeled with colors. Table 6 summarizes the classification results obtained in four validation experiments conducted two days after the calibration of the eNose (using the PCA of Figure 7 as reference data sets for the *k*-NN). The instantaneous classification of the gas detected in Figure 8 was successful in the 94% of the measurements, with an average instantaneous success of 97% in all the experiments conducted in this paper. Therefore, the majority decision of which gas is detected in each experiment was successful in all cases.

Finally, Table 7 summarizes the classification results obtained in four additional validation experiments conducted two weeks after the calibration of the eNose (using the PCA of Figure 7 as reference data sets for the *k*-NN). The classification results show successful instantaneous classification ratios higher than 70% and an average instantaneous success of 77%, so again the majority decision of which gas is detected in each validation experiment has been successful in all cases. However, the comparison of the results of Table 6 and Table 7 evidences that the PCA projection used in the *k*-NN is less representative after two weeks of continuous operation.

## 6. Conclusions

This paper proposes the development and test of a compact and portable eNose based on the use of 16 single-type miniature MEMS MOX gas sensors operating at different heating temperatures. The eNose presented in this paper has been implemented on a single PCB of 80.87 mm × 40.01 mm using a low-power ARM microcontroller and 16 miniature BME680 sensors capable of performing simultaneous measures of air temperature, pressure, humidity and also the total volatile organic compounds that are measured with an embedded MEMS MOX gas sensor. The maximum power consumption of this eNose implementation is 0.9 W (5.0 V, 180 mA) and the standby power is 0.05 W (5.0 V, 10 mA), making it ideal for portable and battery-based applications.

The main disadvantage of common MOX gas sensors are crossed sensitivity to different gases and different crossed sensitivities. This eNose proposal increases these sensor differences by applying different target heater temperature to the 16 MOX gas sensors. Then, the inherent variability and unspecificity that must be expected from the 16 MOX gas sensors, combined with signal processing, are exploited to classify two target volatiles: ethanol and acetone. The proposed eNose reads the resistance of the sensing layer of the 16 embedded MOX gas sensors, applies PCA for dimensional reduction and *k*-NN for classification.

The validation experiments presented in this paper have shown that two individual target volatiles can be detected and classified with an instantaneous classification success higher than 94% two days after the calibration of the eNose and higher than 70% two weeks after this initial calibration. As a summary, in all validation experiments conducted within this period with the proposed eNose, the majority classification of the two target volatiles based on the sequence of instantaneous classification results (threshold > 50%) has been always successfully in controlled laboratory conditions.

The future works planned are mainly the evaluation and improvement of the eNose as a time-varying instrument, such as the analysis of the effect of relative humidity on the sensing in long-term operation, the analysis of the effect of some reducing and oxidizing gases in long-term operation, the refinement of the classificatory and the improvement of the classification performances, and also the application of the eNose in mobile robots as early gas leak detector and as air quality supervisor in unstructured environments.

## Figures and Tables

**Table 2:** BME680 main configuration parameters.

Parameters		Register Name<bit>	Register Values and/or Range	
(1)	Oversampling (T)	*Ctrl_meas*<7:5>	0: Skipped 1: OSx1 2: OSx2	3: OSx4 4: OSx8 5: OSx16
	Oversampling (P)	*Ctrl_hum*<4:2>		
	Oversampling (H)	*Ctrl_hum*<2:0>		
(2)	IIR filter coefficient (T-P)	*Config*<4:2>	0: coefficient = 0 1: coefficient = 1 2: coefficient = 3 3: coefficient = 7	4: coefficient = 15 5: coefficient = 31 6: coefficient = 63 7: coefficient = 127
(3)	Heater off (G)	*Ctrl_gas_0*<3>	1: Off–0: On	
	Enable gas conversion (G)	*Ctrl_1*<4>	1: On–0: Off	
	Heat up duration (G)	*Gas_wait_x*<7:0>	Value representing from 1 ms to 4032 ms	
	Target heater temperature (G)	*Res_heat_x*<7:0>	Value representing from 200 °C to 400 °C	

**Table 3:** List of the combinations of the values of the parameter *gasRange*, and values of const_array1 and const_array2 that must be used during the computation of the resistance of the sensing layer of the MOX gas senor (gas_res, Equation (2)), values provided by the manufacturer.

gasRange	const_array1 Value	const_array2 Value
0	1	8,000,000
1	1	4,000,000
2	1	2,000,000
3	1	1,000,000
4	1	499,500.4995
5	0.99	248,262.1648
6	1	125,000
7	0.992	63,004.03226
8	1	31,281.28128
9	1	15,625
10	0.998	7812.5
11	0.995	3906.25
12	1	1953.125
13	0.99	976.5625
14	1	488.28125
15	1	244.140625

**Table 4:** Configuration of the individual parameters of a BME680 used as a gas sensor.

Parameters	Register Values
Heater off (G)	0: Heater On
Enable gas conversion (G)	1: Run Gas
Heat up duration (G)	Value from 1 ms to 4032 ms
Target heater temperature (G)	Value from 200 °C to 400 °C

**Table 5:** Configuration of the parameters of the 16 BME680 gas sensors used in the eNose.

Sensor ID	Target Heater Temperature (°C)	Heat Up Duration (ms)
1	200	150
2	212	150
3	224	150
4	240	150
5	250	150
6	260	150
7	280	150
8	300	150
9	320	150
10	330	150
11	340	150
12	350	150
13	360	150
14	370	150
15	380	150
16	400	150

**Table 6:** Classifier results obtained when performing experiments two days after calibration.

Experiment	Volatile	Classifier Output (%)		Number of Samples			Success Rate (%)
		Ethanol	Acetone	Total	Hit	Miss	
-	Ethanol	100.00%	0.00%	4073	4073	0	100.00%
Figure 8a	Ethanol	94.90%	5.10%	6215	5898	317	94.90%
-	Acetone	0.00%	100.00%	1971	1971	0	100.00%
Figure 8b	Acetone	3.03%	96.97%	2047	1985	62	96.97%
Average				14,306	13,927	379	97.35%

**Table 7:** Classifier results obtained when performing experiments two weeks after calibration.

Experiment	Volatile	Classifier Output (%)		Number of Samples			Success Rate (%)
		Ethanol	Acetone	Total	Hit	Miss	
-	Ethanol	70.65%	29.35%	4201	2968	1233	70.65%
-	Ethanol	70.95%	29.05%	3349	2376	973	70.95%
-	Acetone	9.24%	90.76%	4046	3672	374	90.76%
-	Acetone	25.48%	74.52%	3375	2515	860	74.52%
Average				14,971	11,531	3440	77.02%

## Data Availability

Not applicable.

## References

[1] Ampuero S., Bosset J.O. (2003). The electronic nose applied to diary products: A review. Sens. Actuators B Chem..

[2] Sarig Y. (2000). Potential applications of artificial olfactory sensing for quality evaluation of fresh produce. J. Agric. Eng. Res..

[3] Covington J.A., Marco S., Persaud K.C., Schiffman S.S., Nagle H.T. (2021). Artificial Olfaction in the 21st Century. IEEE Sens. J..

[4] Westenbrink E., Arasaradnam R.P., O’Connell N., Bailey C., Nwokolo C. (2015). Development and application of a new electronic nose instrument for the detection of colorectal cancer. Biosens. Bioelectron..

[5] Wilson A.D. (2013). Diverse Applications of Electronic-Nose Technologies in Agriculture and Forestry. Sensors.

[6] So S., Koushanfar F., Kosterev A., Tittel F. LaserSPECks: Laser SPECtroscopic Trace-Gas Sensor Networks—Sensor Integration and Applications. Proceedings of the 6th International Conference on Information Processing in Sensor Networks.

[7] Wang D., Agrawal D.P., Toruksa W., Chaiwatpongsakorn C., Lu M., Keener T.C. (2010). Monitoring ambient air quality with carbon monoxide sensor-based wireless network. Commun. ACM.

[8] Rossi M., Brunelli D. Analyzing the transient response of MOX gas sensors to improve the lifetime of distributed sensing systems. Proceedings of the 5th IEEE International Workshop on Advances in Sensors and Interfaces (IWASI).

[9] Chiu S.-W., Tang K.-T. (2013). Towards a Chemiresistive Sensor-Integrated Electronic Nose: A Review. Sensors.

[10] Zampolli S., Elmi I., Ahmed F., Passini M., Cardinali G.C., Nicoletti S., Dori L. (2004). An electronic nose based on solid state sensor arrays for low-cost indoor air quality monitoring applications. Sens. Actuators B Chem..

[11] Bhattacharya S., Sridevi S., Pitchiah R. Indoor Air Quality Monitoring using Wireless Sensor Network. Proceedings of the 2012 Sixth International Conference on Sensing Technology (ICST).

[12] Song K., Wang Q., Liu Q., Zhang H., Cheng Y. (2011). A wireless electronic nose system using a Fe_2_O_3_ gas sensing array and least squares support vector regression. Sensors.

[13] Fine G.F., Cavanagh L.M., Afonja A., Binions R. (2010). Metal oxide semi-conductor gas sensors in environmental monitoring. Sensors.

[14] Wetchakun K., Samerjai T., Tamaekong N., Liewhiran C., Siriwong C., Kruefu V., Wisitsoraat A., Tuantranont A., Phanichphant S. (2011). Semiconducting metal oxides as sensors for environmentally hazardous gases. Sens. Actuators B Chem..

[15] Ghasemi-Varnamkhasti M., Mohtasebi S.S., Siadat M., Balasubramanian S. (2009). Meat Quality Assessment by Electronic Nose (Machine Olfaction Technology). Sensors.

[16] Peris M., Escuder-Gilabert L. (2009). A 21st century technique for food control: Electronic noses. Anal. Chim. Acta.

[17] Gardner J.W., Shin H.W., Hines E.L. (2000). An electronic nose system to diagnose illness. Sens. Actuators B Chem..

[18] Nakhleh M.K., Amal H., Jeries R., Broza Y.Y., Aboud M., Gharra A., Ivgi H., Khatib S., Badarneh S., Har-Shai L. (2017). Diagnosis and Classification of 17 Diseases from 1404 Subjects via Pattern Analysis of Exhaled Molecules. ACS Nano.

[19] Liu H., Zhang L., Li K.H.H., Tan O.K. (2018). Microhotplates for Metal Oxide Semiconductor Gas Sensor Applications—Towards the CMOS-MEMS Monolithic Approach. Micromachines.

[20] Meixner H., Lampe U. (1996). Metal oxide sensors. Sens. Actuators B Chem..

[21] Wang C., Yin L., Zhang L., Xiang D., Gao R. (2010). Metal Oxide Gas Sensors: Sensitivity and Influencing Factors. Sensors.

[22] Eranna G., Joshi B.C., Runthala D.P., Gupta R.P. (2010). Oxide Materials for Development of Integrated Gas Sensors—A Comprehensive Review. Crit. Rev. Solid State Mater. Sci..

[23] Ali S., Gupta A., Shafiei M., Langford S.J. (2021). Recent Advances in Perylene Diimide-Based Active Materials in Electrical Mode Gas Sensing. Chemosensors.

[24] Choi J.H., Lee J., Byeon M., Hong T.E., Park H., Lee C.Y. (2020). Graphene-Based Gas Sensors with High Sensitivity and Minimal Sensor-to-Sensor Variation. ACS Appl. Nano Mater..

[25] Persaud K., Dodd G. (1982). Analysis of discrimination mechanisms in the mammalian olfactory system using a model nose. Nature.

[26] Marco S., Ortega A., Pardo A., Samitier J. (1998). Gas identification with tin oxide sensor array and self-organizing maps: Adaptive correction of sensor drifts. IEEE Trans. Instrum. Meas..

[27] Kohonen T. (1982). Self-organized formation of topologically correct feature maps. Biol. Cybern..

[28] Arnold C., Harms M., Goschnick J. (2002). Air quality monitoring and fire detection with the Karlsruhe electronic micronose KAMINA. IEEE Sens. J..

[29] Bennetts V.H., Lilienthal A.J., Trincavelli M. Creating true gas concentration maps in presence of multiple heterogeneous gas sources. Proceedings of the 2012 IEEE Sensors.

[30] Marco S., Gutiérrez-Gálvez A., Lansner A., Martinez D., Rospars J.P., Beccherelli R., Perera A., Pearce T.C., Verschure P.F.M.J., Persaud K. (2014). A biomimetic approach to machine olfaction, featuring a very large-scale chemical sensor array and embedded neuro-bio-inspired computation. Microsyst Technol.

[31] Burgués J., Jiménez-Soto J.M., Marco S. (2018). Estimation of the limit of detection in semiconductor gas sensors through linearized calibration models. Anal. Chim. Acta.

[32] Burgués J., Marco S. (2018). Low Power Operation of Temperature-Modulated Metal Oxide Semiconductor Gas Sensors. Sensors.

[33] Rossi M., Brunelli D. (2016). Autonomous Gas Detection and Mapping with Unmanned Aerial Vehicles. IEEE Trans. Instrum. Meas..

[34] Palacín J., Martínez D., Clotet E., Pallejà T., Burgués J., Fonollosa J., Pardo A., Marco S. (2019). Application of an Array of Metal-Oxide Semiconductor Gas Sensors in an Assistant Personal Robot for Early Gas Leak Detection. Sensors.

[35] Burgués J., Deseada-Esclapez M., Doñate S., Marco S. (2021). RHINOS: A lightweight portable electronic nose for real-time odor quantification in wastewater treatment plants. iScience.

[36] Bishop C.M. (2006). Pattern Recognition and Machine Learning (Information Science and Statistics).

[37] Wold S., Ruhe A., Wold H., Dunn W.J. (1984). The Collinearity Problem in Linear Regression. The Partial Least Squares (PLS) Approach to Generalized Inverses. SIAM J. Sci. Stat. Comput..

[38] Arroyo P., Meléndez F., Suárez J.I., Herrero J.L., Rodríguez S., Lozano J. (2020). Electronic Nose with Digital Gas Sensors Connected via Bluetooth to a Smartphone for Air Quality Measurements. Sensors.

[39] Gardner M.W., Dorling S.R. (1998). Artificial neural networks (the multilayer perceptron)—A review of applications in the atmospheric sciences. Atmos. Environ..

[40] Burgués J., Hernández V., Lilienthal A.J., Marco S. (2020). Gas distribution mapping and source localization using a 3D grid of metal oxide semiconductor sensors. Sens. Actuators B Chem..

[41] Tiele A., Wicaksono A., Ayyala S.K., Covington J.A. (2020). Development of a Compact, IoT-Enabled Electronic Nose for Breath Analysis. Electronics.

[42] Ballabio D., Consonni V. (2013). Classification tools in chemistry. Part 1: Linear models. PLS-DA. Anal. Methods.

[43] Fan H., Hernandez Bennetts V., Schaffernicht E., Lilienthal A.J. (2019). Towards Gas Discrimination and Mapping in Emergency Response Scenarios Using a Mobile Robot with an Electronic Nose. Sensors.

[44] Wold S., Sjöström M., Eriksson L. (2001). PLS-regression: A basic tool of chemometrics. Chemom. Intell. Lab. Syst..

[45] Gongora A., Chaves D., Jaenal A., Monroy J., Gonzalez-Jimenez J. (2018). Toward the generation of smell maps: Matching electro-chemical sensor information with human odor perception. Front. Artif. Intell. Appl..

[46] Monroy J.G., Gonzalez-Jimenez J. (2017). Gas classification in motion: An experimental analysis. Sens. Actuators B Chem..

[47] Schleif F.M., Hammer B., Monroy J.G., Jimenez J.G., Blanco-Claraco J.L., Biehl M., Petkov N. (2016). Odor recognition in robotics applications by discriminative time-series modeling. Pattern Anal. Appl..

[48] Fonollosa J., Fernández L., Gutiérrez-Gálvez A., Huerta R., Marco S. (2016). Calibration transfer and drift counteraction in chemical sensor arrays using Direct Standardization. Sens. Actuators B Chem..

[49] Vapnik V. (1995). The Nature of Statistical Learning Theory.

[50] Vries R., Brinkman P., van der Schee M.P., Fens N., Dijkers E., Bootsma S.K., de Jongh F.H.C., Sterk P.J. (2015). Integration of electronic nose technology with spirometry: Validation of a new approach for exhaled breath analysis. J. Breath Res..

[51] González-Arjona D., González A.G. (1998). Adaptation of linear discriminant analysis to second level-pattern recognition classification. Anal. Chim. Acta.

[52] Fonollosa J., Sheik S., Huerta R., Marco S. (2015). Reservoir computing compensates slow response of chemosensor arrays exposed to fast varying gas concentrations in continuous monitoring. Sens. Actuators B Chem..

[53] Natschläger T., Maass W., Markram H. (2002). The “Liquid Computer”: A Novel Strategy for Real-Time Computing on Time Series. Telematik.

[54] Rossi M., Brunelli D., Adami A., Lorenzelli L., Menna F., Remondino F. Gas-Drone: Portable gas sensing system on UAVs for gas leakage localization. Proceedings of the 2014 IEEE SENSORS.

[55] Sanchez-Garrido C., Monroy J.G., Gonzalez-Jimenez J. A configurable smart e-nose for spatio-temporal olfactory analysis. Proceedings of the 2014 IEEE SENSORS.

[56] Monroy J.G., Gonzalez-Jimenez J., Sanchez-Garrido C. Monitoring household garbage odors in urban areas through distribution maps. Proceedings of the 2014 IEEE Sensors.

[57] Bennetts V.H., Schaffernicht E., Pomareda V., Lilienthal A.J., Marco S., Trincavelli M. (2014). Combining Non Selective Gas Sensors on a Mobile Robot for Identification and Mapping of Multiple Chemical Compounds. Sensors.

[58] Savarese M., Caporaso N., Parisini C. Application of an Electronic Nose for the Evaluation of Rancidity and Shelf live in Virgin Olive Oil. Proceedings of the Electronic International Interdisciplinary Conference (EIIC 2013).

[59] Monroy J.G., Lilienthal A.J., Blanco J.-L., Gonzalez-Jimenez J., Trincavelli M. (2013). Probabilistic gas quantification with MOX sensors in Open Sampling Systems—A Gaussian Process approach. Sens. Actuators B Chem..

[60] Vergara A., Vembu S., Ayhan T., Ryan M.A., Homer M.L., Huerta R. (2012). Chemical gas sensor drift compensation using classifier ensembles. Sens. Actuators B Chem..

[61] Thayananthan A., Navaratnam R., Stenger B., Torr P.H.S., Cipolla R. Multivariate Relevance Vector Machines for Tracking. Proceedings of the Computer Vision–ECCV 2006.

[62] Aguilera T., Lozano J., Paredes J.A., Álvarez F.J., Suárez J.I. (2012). Electronic Nose Based on Independent Component Analysis Combined with Partial Least Squares and Artificial Neural Networks for Wine Prediction. Sensors.

[63] Jutten C., Herault J. (1991). Blind separation of sources, part I: An adaptive algorithm based on neuromimetic architecture. Signal Processing.

[64] Brudzewski K., Osowski S., Dwulit A. (2012). Recognition of Coffee Using Differential Electronic Nose. IEEE Trans. Instrum. Meas..

[65] Haddi Z., Amari A., Alami H., El Bari N., Llobet E., Bouchikhi B. (2011). A portable electronic nose system for the identification of cannabis-based drugs. Sens. Actuators B Chem..

[66] Gonzalez-Jimenez J., Monroy J.G., Blanco J.L. (2011). The Multi-Chamber Electronic Nose—An Improved Olfaction Sensor for Mobile Robotics. Sensors.

[67] Brudzewski K., Osowski S., Ulaczyk J. (2010). Differential electronic nose of two chemo sensor arrays for odor discrimination. Sens. Actuators B Chem..

[68] Guo D., Zhang D., Li N., Zhang L., Yang J. (2010). A Novel Breath Analysis System Based on Electronic Olfaction. IEEE Trans. Biomed. Eng..

[69] Mildner-Szkudlarz S., Jenen H.H. (2010). Detection of olive oil adulteration with rapeseed and sunflower oils using MOS electronic nose and SPME-MS. J. Food Qual..

[70] Lilienthal A.J., Reggente M., Trincavelli M., Blanco J.L., Gonzalez J. A statistical approach to gas distribution modelling with mobile robots—The Kernel DM+V algorithm. Proceedings of the 2009 IEEE/RSJ International Conference on Intelligent Robots and Systems.

[71] Pearson K. (1901). On Lines and Planes of Closest Fit to Systems of Points in Space. Lond. Edinb. Dublin Philos. Mag. J. Sci..

[72] Fix E., Hodges J.L. (1951). Discriminatory Analysis. Nonparametric Discrimination: Consistency Properties.

